# Chromium‐Induced High Covalent Co–O Bonds for Efficient Anodic Catalysts in PEM Electrolyzer

**DOI:** 10.1002/advs.202402356

**Published:** 2024-04-22

**Authors:** Qisheng Yan, Jie Feng, Wenjuan Shi, Wenzhe Niu, Zhuorong Lu, Kai Sun, Xiao Yang, Liangyao Xue, Yi Liu, Youyong Li, Bo Zhang

**Affiliations:** ^1^ State Key Laboratory of Molecular Engineering of Polymers Department of Macromolecular Science Fudan University Shanghai 200438 China; ^2^ Institute of Functional Nano & Soft Materials (FUNSOM) and Jiangsu Key Laboratory for Carbon‐Based Functional Materials & Devices Soochow University Suzhou 215123 China

**Keywords:** cobalt oxides, non‐precious metal catalysts, oxygen evolution reaction, proton exchange membrane water electrolyzer

## Abstract

The proton exchange membrane water electrolyzer (PEMWE), crucial for green hydrogen production, is challenged by the scarcity and high cost of iridium‐based materials. Cobalt oxides, as ideal electrocatalysts for oxygen evolution reaction (OER), have not been extensively applied in PEMWE, due to extremely high voltage and poor stability at large current density, caused by complicated structural variations of cobalt compounds during the OER process. Thus, the authors sought to introduce chromium into a cobalt spinel (Co_3_O_4_) catalyst to regulate the electronic structure of cobalt, exhibiting a higher oxidation state and increased Co–O covalency with a stable structure. In‐depth operando characterizations and theoretical calculations revealed that the activated Co–O covalency and adaptable redox behavior are crucial for facilitating its OER activity. Both turnover frequency and mass activity of Cr‐doped Co_3_O_4_ (CoCr) at 1.67 V (vs RHE) increased by over eight times than those of as‐synthesized Co_3_O_4_. The obtained CoCr catalyst achieved 1500 mA cm^−2^ at 2.17 V and exhibited notable durability over extended operation periods – over 100 h at 500 mA cm^−2^ and 500 h at 100 mA cm^−2^, demonstrating promising application in the PEMWE industry.

## Graphical abstract

This research reports a promising non‐precious metal anodic electrocatalyst for a proton exchange membrane water electrolyzer (PEMWE). Owing to high covalent Co–O bonds induced by Cr, the CoCr electrocatalyst exhibits notable durability over extended operation periods, over 100 h at 500 mA cm^−2^ and 500 h at 100 mA cm^−2^, demonstrating considerable application potential in PEMWE devices.

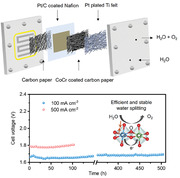

## 1 Introduction

Hydrogen is not only a green and sustainable energy vector but also a prospective energy storage carrier, capable of tackling the challenges posed by intermittent power production connected with wind, solar, and other time and/or space‐variable sources.^[^ ^1^ ^]^ Proton exchange membrane water electrolyzer (PEMWE) has garnered attention due to its superior characteristics over alkaline electrolyzers, including lower resistance losses, higher current density, enhanced hydrogen (H_2_) purity, and larger partial‐load range.^[^ ^1^, ^2^ ^]^ However, oxygen evolution reaction (OER) occurring at the anodic side of the PEMWE requires a higher overpotential (η) owing to its complex electron transfer mechanism,^[^ ^3^ ^]^ predominantly relying on precious metal‐based materials like IrO_2_ and RuO_2_ for their activity and stability under acidic conditions (pH 2–4 in the local environment of the PEMWE).^[^ ^2^, ^4^ ^]^ It is concerning that the abundance of iridium on Earth is merely 0.001 ppm, approximately one‐fortieth of the gold content, severely impeding the widespread application feasibility of the PEMWE.^[^ ^5^ ^]^ Although efforts to reduce iridium loading have been made,^[^ ^6^ ^]^ the ultimate solution hinges on developing non‐precious metal catalysts to serve as substitutes for precious metal‐based catalysts.

As known, Co_3_O_4_ with Co^2+^ occupied in tetrahedral (T_d_) sites and Co^3+^ in octahedral (O_h_) sites, is predicted as a promising acidic OER catalyst with high activity and stability by density functional theory (DFT) calculations and Pourbaix diagram analysis.^[^ ^2^, ^7^ ^]^ Nevertheless, Co species go through redox transitions, typically between Co^2+/3+^ and Co^3+/4+^ during the OER process, which easily causes the delayed onset of OER.^[^ ^8^ ^]^ Previous studies have focused on improving OER activity by creating defects (such as oxygen vacancies) to accelerate the reconstruction process and tune the adsorption‐free energy,^[^ ^9^ ^]^ but the defects usually disrupt the structure, especially at large current density in acidic media,^[^ ^10^ ^]^ affecting the usage of the catalysts in PEMWE. Inducing strong Metal(M)‐O Covalent raises the possibility of electronic transition to promote the formation of intermediates,^[^ ^11^ ^]^ becoming a potential alternative strategy. Meanwhile, the enhanced M–O covalency seems to suppress metal leaching out and improve structural stability.^[^ ^12^ ^]^

**Figure 1:**
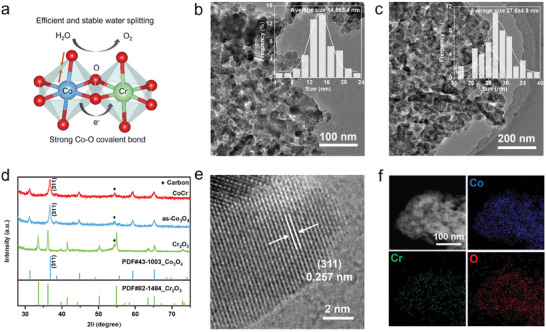
a) Design concept of the efficient anodic catalyst for PEMWE. b,c) TEM images and corresponding particle size distribution of b) CoCr and c) as‐Co_3_O_4_, showing a smaller size in the CoCr catalyst. d) XRD patterns of the catalysts, indicating the spinel structure of Co_3_O_4_. The dominant peak at about 36.8° refers to (311) surface. e) HRTEM image of CoCr catalyst. f) Dark‐field TEM images and relevant EDS mapping of Co, Cr, and O in CoCr catalyst. All the elements are dispersed uniformly.

VIB group cations possess a large number of vacant *d*‐orbitals, which can efficiently modulate the electronic structure of Co and promote alkaline OER.^[^ ^13^ ^]^ Chromium (Cr), with multiple oxidation states, presumably can be utilized to facilitate the generation of the active sites.^[^ ^9^, ^14^ ^]^ Moreover, doping Cr is beneficial for regulating the filling state of e_g_ electrons of transition metals (TM), contributing to the hybridization between TM 3d‐O 2p,^[^ ^15^ ^]^ which is generally relevant with a high degree of TM–O covalency. We propose that introducing Cr into Co_3_O_4_ can optimize the ratio of Co^2+^ and Co^3+^, enhance the covalency of Co–O bonds, and accelerate the pre‐oxidation process, thereby achieving low voltage and high stability in PEMWE (**Figure** **1**a).

Herein, we sought to synthesize an expected Cr‐doped (10% mol) Co_3_O_4_ (CoCr) via polymer‐assisted in situ growth method on carbon paper (CP), as a promising anodic electrocatalytic material for PEMWE. Operando X‐ray adsorption near‐edge structures (XANES) and DFT results indicate that after introducing Cr, the Co–O covalency is indeed enlarged, and stimulates electron transfer between Co cations and oxygen adsorbates. Further operando Raman spectroscopy studies show that active Co^4+^ species are generated at a lower potential in CoCr than that in the pristine Co_3_O_4_, which was synthesized via the same method without Cr adding. The quick Co^3+/4+^ redox response owing to the more covalent and flexible Co–O bonds, further breaks the activity/stability trade‐off. The CoCr electrocatalyst achieved 1500 mA cm^−2^ at 2.17 V and exhibited notable durability over extended operation periods, over 100 h at 500 mA cm^−2^ and 500 h at 100 mA cm^−2^, demonstrating considerable application potential in PEMWE devices. This achievement is particularly noteworthy as practical applications of most non‐precious metal catalysts have not been extensively reported until now.

## 2 Results and Discussion

**Scheme 1:**
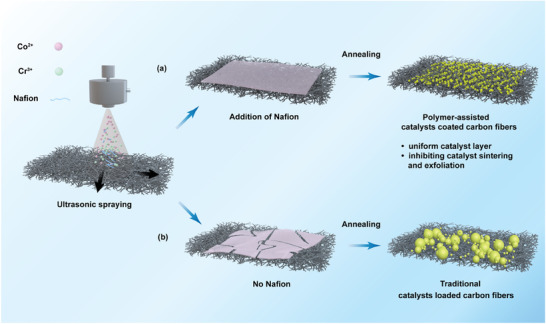
Proposed strategy to synthesize the catalysts on carbon paper. a) Polymer‐assisted catalysts coated carbon fibers. b) Traditional catalysts loaded carbon fibers without Nafion.

CoCr and as‐synthesized Co_3_O_4_ (as‐Co_3_O_4_) catalysts were synthesized directly on CP by spraying the corresponding metal nitrate precursors and Nafion, followed by annealing in air (**Scheme** **1**, see Supporting Information for details). The traditional method is to only drop the precursor aqueous on the substrate,^[^ ^16^ ^]^ facing the inevitable rough surface and inhomogeneous layer during the evaporation process caused by surface tension. Inspired by the preparation of catalyst gas diffusion electrode, we added perfluorosulfonic acid polymer (Nafion) as a binder into the aqueous to form a uniform layer tightly wrapping carbon fibers, avoiding issues of catalyst sintering and exfoliation during the annealing process and improve catalytic activity (Figure S1, Supporting Information). X‐ray diffraction (XRD) patterns reveal the Co_3_O_4_ spinel structure in the CoCr catalyst (Figure 1d). Notably, the (311) peaks shifted to a lower degree with higher Cr doping, suggesting an expansion in interplanar spacing as high‐resolution transmission electron microscopy (HRTEM) showed (Figure 1d,e, and Figure S2, Supporting Information). Interestingly, no Cr_2_O_3_ phase was detected in the materials, except when chromic nitrate alone was used in the synthesis process. TEM and scanning electron microscopy (SEM) images showed that the CoCr nanoparticles have a normal distribution with an average diameter of ≈14.6 nm, which is nearly half the size of the as‐Co_3_O_4_ nanoparticles (Figure 1b,c, and Figure S3, Supporting Information). Energy dispersive X‐ray spectroscopy (EDS) confirmed that the atomic ratio of elements in the obtained CoCr materials is 8.4, which is consistent with inductively coupled plasma optical emission spectrometry (ICP‐OES) results, and all the elements are dispersed homogeneously in nanoparticles (Figure 1f, and Figure S4 and Table S1, Supporting Information).

**Figure 2:**
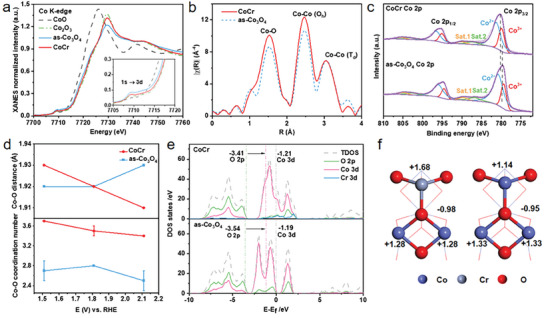
Characterization of electronic structures. a) XANES spectra of standard and as‐synthesized samples at Co K‐edge. Inset enlarges the pre‐edge 1s → 3d transition and illustrates a right‐shifted absorption energy and a decreased pre‐edge peak intensity in CoCr catalysts, implying a higher Co oxidation state and local geometry of the Co ions. b) Fourier transforms (k^3^‐weighted) of Co K‐edge EXAFS spectra for CoCr and as‐Co_3_O_4_ catalysts at open circuit potential (OCP). The three characteristic peaks represent the paths of Co‐O, Co–Co octahedral (O_h_), and Co–Co tetrahedral (T_d_), respectively. c) XPS spectra of Co 2p. XPS fitting results reveal that CoCr exhibits a higher Co^3+^/Co^2+^ ratio of 0.49 than as‐Co_3_O_4_ (0.36) in accordance with XANES spectra. d) Co‐O bond distance and coordination number (CN) derived from EXAFS fitting data measured at different potentials (vs. RHE). e) Computed total electronic density of states (TDOS) and partial electronic density of states (PDOS) of CoCr and as‐Co_3_O_4_. The green, pink, and grey dotted lines represent the center of O 2p, Co 3d band, and Fermi level (E_f_), respectively. f) The calculated Bader charge of Co, Cr, and O in CoCr and as‐Co_3_O_4_.

To clarify the electronic structure and the coordination environment of the catalysts, we performed synchrotron X‐ray absorption spectroscopy (XAS) and X‐ray photoelectron spectroscopy (XPS). XANES curves of CoO (T_d_, a t_2g_^5^e_g_^2^ electron configuration) and Co_2_O_3_ (O_h_, a t_2g_^6^e_g_^0^ electron configuration) were measured as references.^[^ ^17^ ^]^ Firstly, the XANES spectrum at the Co K‐edge of CoCr closely resembles that of Co_2_O_3_ with a higher white line (WL) intensity compared to that of as‐Co_3_O_4_. This suggests a higher coordination number (CN) of the Co–O bonds in the CoCr catalyst (**Figure** **2**a).^[^ ^18^ ^]^ Secondly, the Co K‐edge curves of CoCr display a positive edge shift, indicative of a higher average oxidation state, a result corroborated by XPS fitting data (Figure 2a,c).^[^ ^19^ ^]^ Additionally, the weakened intensity of the pre‐edge peak (1s → 3d transition) in CoCr suggests an increase in the local symmetry of cobalt ions (Figure 2a).^[^ ^19^, ^20^ ^]^ The enhanced symmetry is also confirmed by O K‐edge curves (Figure S5, Supporting Information). Thirdly, the extended X‐ray absorption fine structure (EXAFS) spectra with Fourier transforms (k^3^‐weighted) Co K‐edge of both CoCr and as‐Co_3_O_4_ catalysts at open circuit potential (OCP) possesses three major signals representing Co–O, Co–Co (O_h_), and Co–Co (T_d_) scattering paths (Figure 2b).^[^ ^21^ ^]^ The intensity of the Co–O first shell for the CoCr sample increases, which is in accordance with the larger CN of the Co–O bond in the fitting results (Tables S2 and S3, Supporting Information). The XPS results show that the binding energy of Co 2p_3/2_ in the CoCr sample slightly shifts in a higher direction relative to as‐Co_3_O_4_, probably caused by electron transfer and electronic coupling between Co and Cr (Figure 2c).^[^ ^22^ ^]^ The Cr 2p spectrum displays two peaks corresponding to Cr 2p_1/2_ and Cr 2p_3/2_ core levels, respectively (Figure S6, Supporting Information). The Cr 2p_3/2_ peak is further deconvoluted into two components at 577.6 and 576.2 eV, which can be attributed to Cr^3+^–OH and Cr^3+^–O, respectively.^[^ ^23^ ^]^

We conducted DFT calculations to deepen our understanding of how Cr regulates the electronic structure of Co, including total electronic density of states (TDOS) and partial electronic density of states (PDOS) calculations for both CoCr and as‐Co_3_O_4_ (Figure 2e). These calculations revealed the presence of the lowest conduction band and a valence band spanning a range of 10 eV. The densities of states of as‐Co_3_O_4_ at the Fermi level (E_f_) are negligible, but those of CoCr become incredibly strong, meaning better conductivity.^[^ ^11d^ ^]^ We found that the introduction of Cr resulted in an enhancement of the O 2p band center and a reduction in the energy difference between the Co 3d and O 2p band centers, suggesting an increase in Co–O covalency. This was further determined by the decreased charge difference between Co and O in CoCr relying on Bader charge analysis (Figure 2f). This heightened Co–O covalency can favor the electron transfer between Co cation and oxygen adsorbates, thereby potentially facilitating the OER process.^[^ ^11c^ ^]^

**Figure 3:**
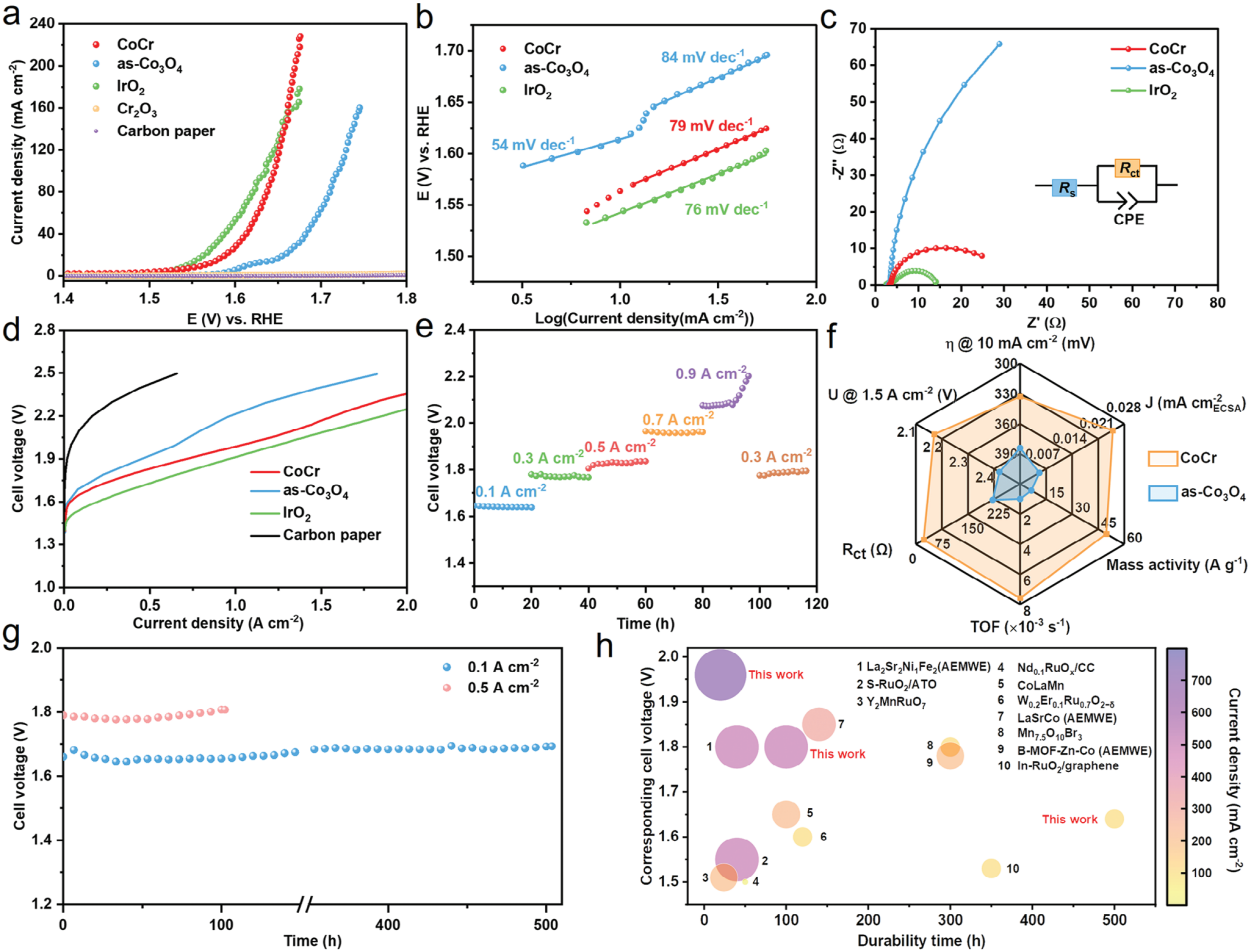
Electrochemical performance of catalysts. a) LSV of catalysts collected at a 5 mV s^−1^ scan rate in 0.5 M H_2_SO_4_ with iR correction. b) Tafel plots analysis. There are two distinct regions in as‐Co_3_O_4_ catalysts due to the oxidation of Co^3+^ to Co^4+^. c) EIS measurements recorded at 1.57 V versus RHE. d) Current‐voltage polarizations of catalysts in PEWME. e) Chronopotentiometry curves of the PEMWE using sequential current densities. f) Comparison of electrocatalytic metrics between CoCr and as‐Co_3_O_4_. The ECSA‐normalized current density, mass activity, and TOF data were provided at 1.67 V versus RHE. The PEMWE voltage (U) was obtained at 1.5 A cm^−2^. *R_ct_* was selected from EIS fitting results. g) Chronopotentiometry curves of the PEMWE using CoCr operated at 0.1 and 0.5 A cm^−2^ at 80 °C with Nafion 212 membrane. h) Comparison of membrane electrode assembly (MEA) performance for precious reported electrocatalysts (Table S6, Supporting Information). Both the size of bubble and the shade of color represent the magnitude of the current density. The *x‐* and *y*‐axes refer to the longest durable time and corresponding cell voltage, respectively. High current density is considered as one of the most important indicators for measuring the performance of MEA. The electrocatalysts marked AEMWE are applied in the anion exchange membrane water electrolyzer.

The electrocatalytic activity of the CoCr catalyst was assessed within a three‐electrode system in 0.5 M H_2_SO_4_ solution. The results, based on the linear sweep voltammetry (LSV) curves, revealed varying degrees of overpotential reduction upon the introduction of different contents of Cr. Among different doping contents, 10% Cr‐doped (mol%) exhibited optimal performance (Figure S7, Supporting Information). The overpotential of CoCr at 10 mA cm^−2^ was only 333 mV, much smaller than that of as‐Co_3_O_4_ (385 mV) (**Figure** **3**a). Commercial IrO_2_, always used as a benchmark acidic OER catalyst, exhibited an overpotential of 314 mV@10 mA cm^−2^, and Cr_2_O_3_ and carbon paper showed negligible electrocatalytic activity. To gain further insights into the kinetics of OER, we calculated Tafel slopes for CoCr and as‐Co_3_O_4_, resulting in values of 79 and 84 mV dec^−1^, respectively (Figure 3b), which shows a mixed kinetic control mechanism in the range of 60–120 mV dec^−1^.^[^ ^24^ ^]^ Additionally, another linear Tafel region was found in as‐Co_3_O_4_, which is connected to Co^3+/4+^ redox peaks observed in cyclic voltammetry (CV) curves according to the previous report (Figure S8, Supporting Information), suggesting a sluggish charge accumulation process.^[^ ^21^ ^]^ The electrochemical impedance spectroscopy (EIS) studies suggest that the incorporation of Cr led to a remarkable reduction in charge‐transfer resistance (R_ct_) from 221 to 22.7 Ω (Figure 3c, and Table S4, Supporting Information), indicating a higher catalytic activity. The performances of some previous non‐iridium‐based electrocatalysts in acid were listed for comparison (Table S5, Supporting Information).

To highlight the superiority of the CoCr electrocatalyst, we displayed electrocatalytic metrics with six dimensions in the radar chart (Figure 3f). The electrochemically active surface area (ECSA) was measured by analyzing the scan rate CV‐dependence plot (Figure S9, Supporting Information). The ECSA‐normalized current density at 1.67 V versus RHE for CoCr was 0.025 mA cm 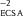, over four times higher than that of as‐Co_3_O_4_ (Figure S10, Supporting Information). The mass‐specific activity of CoCr reached 49.6 A g^−1^ at 1.67 V versus RHE, marking an 8‐fold improvement compared to as‐Co_3_O_4_. In terms of turnover frequency (TOF), CoCr achieved 7.58 × 10^−3^ s^−1^ at 1.67 V versus RHE, representing an 8‐fold enhancement over pristine as‐Co_3_O_4_ (0.99 × 10^−3^ s^−1^). More importantly, when applied in the PEMWE, we observed that the cell voltages required for CoCr and as‐Co_3_O_4_ to reach a current density of 1.5 A cm^−2^ was 2.17 V, much lower than that of as‐Co_3_O_4_ (2.42 V) (Figure 3d).

We then turned to evaluate activity and stability in 0.1 M HClO_4_ solution, which is closer to the real pH in PEMWE (Figures S11 and S12, Supporting Information). The CoCr catalyst still exhibits a lower overpotential of 10 mA cm^−2^ than as‐Co_3_O_4_ in 0.1 M HClO_4_. We used ICP‐OES to monitor the elemental dissolution situation during long‐term chronopotentiometry tests at 10 mA cm^−2^ (Figure S13, Supporting Information). It was observed that Co and Cr leaching rates reached a steady state after ≈10 h. Importantly, we noted that there were no apparent changes in terms of morphology, nanoparticle size, and elemental distribution after OER (Figures S14 and S15, Supporting Information). Furthermore, we carried out a chronopotentiometry test to gain deeper insights into the performance of the CoCr catalyst in PEMWE, incrementally increasing the current density every 20 h (from 0.1 to 0.9 A cm^−2^). Remarkably, there was no visible voltage increase until reaching 0.9 A cm^−2^ and only a 20‐mV increase occurred when shifting back to 0.3 A cm^−2^ (Figure 3e). Therefore, we deem that 900 mA cm^−2^ is the upper limit current density for stable operation of the CoCr catalyst, under which the catalyst will face deactivation issues. Finally, the CoCr catalyst was operated at 0.1 and 0.5 A cm^−2^ steadily for nearly 500 h (1.67 V) and 100 h (1.80 V), respectively, demonstrating its prospective applicability in PEMWE (Figure 3g). The commercial IrO_2_ catalyst was also used as a reference (Figure S16, Supporting Information). To compare with previously reported electrocatalysts in membrane electrode assembly (MEA), we emphasized the magnitude of the current density in a bubble chart since a high current density with excellent MEA stability is extremely desirable (Figure 3h, and Table S6, Supporting Information). It is noted that the CoCr catalyst surpasses most catalysts including some Ru‐based catalysts.

Operando XAS was employed to investigate the structural evolution during the OER process. The average oxidation state of cobalt increased with the rising potential and consistently remained at a higher value in the CoCr compared to the as‐Co_3_O_4_ throughout the entire process (Figure S17, Supporting Information). Simultaneously, Fourier transforms of EXAFS spectra displayed a shortened bond length and reduced intensity of the Co–O shell in the CoCr sample when the potential of 1.51 to 2.11 V was applied (Figure 2d, and Figures S17–S19 and Tables S2 and S3, Supporting Information), which indicates a contraction of Co–O covalency and a reduction in O CN to Co respectively.^[^ ^18^, ^25^ ^]^ Nevertheless, the CN of the Co–O bond decreased only after a potential exceeding 2.11 V and the length of the Co–O bond even increased in as‐Co_3_O_4_ (Figure 2d). The loss of O is associated with the progression of the OER.^[^ ^18^ ^]^ Since the CN of Co also kept higher in CoCr than that in as‐Co_3_O_4_, we infer that enlarged covalency of Co–O bonds mostly arises from Co^3+^–O. Thus, the high‐valence cobalt, induced by the presence of Cr, activates the flexible and adaptive Co–O bonds during the OER process.

**Figure 4:**
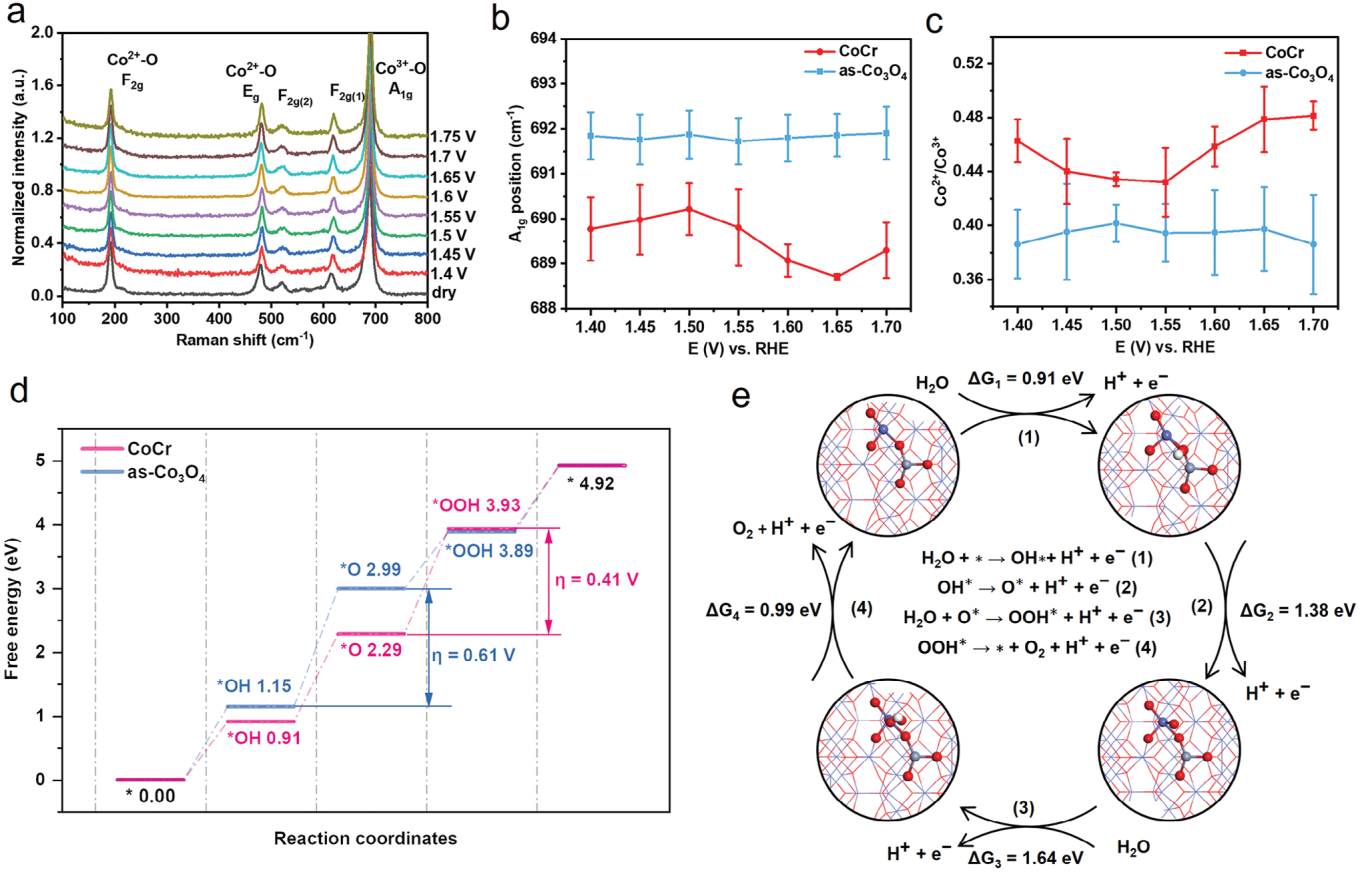
a) Operando Raman spectra of CoCr at various constant potentials (vs RHE) (increasing from 1.4 to 1.75 V). b) The Raman A_1g_ peak positions and c) Co^2+^/Co^3+^ ratio plotted against the applied potential. d) Proposed the OER mechanism on CoCr according to the AEM pathway and the corresponding four‐electron free energy diagram. e) Simulated OER reaction pathways of CoCr with the intermediates adsorbed including OH*, O*, and OOH* on the (311) facet which shows the dominant peak in XRD patterns. The blue, grey, red, and white balls represent Co, Cr, O, and H, respectively. Error bars in (b,c) represent the standard deviation of at least three independent samples.

To get deeper insight into the variations of active sites, a series of operando Raman spectra were obtained at different potentials. It can be obtained that five characteristic Raman peaks belonging to Co_3_O_4_ spinel were observed at about 192, 479, 519, 618, and 690 cm^−1^ (**Figure** **4**a, and Figure S20, Supporting Information), representing F_2g_, E_g_, F_2g(2)_, F_2g(1)_, and A_1g_ vibration modes, respectively.^[^ ^26^ ^]^ The strongest peak A_1g_ was fitted with the Lorentzian function to unravel the evolution of the local bonding environment of the catalysts during the OER process. The A_1g_ peak in CoCr showed a blue shift first and then shifted to the opposite direction after applying a potential of 1.50 V versus RHE, while no obvious shift appeared in as‐Co_3_O_4_ (Figure 4b). The blue shifts often indicate lattice contraction,^[^ ^27^ ^]^ and the red shifts are related to oxidized active sites.^[^ ^21^, ^28^ ^]^ It is reported that the ratio of Co^2+^/Co^3+^ can be estimated by the ratio of the integrated peak intensity of Co^2+^‐O vibrations (F_2g_ and E_g_) to that of Co^3+^‐O vibration (A_1g_).^[^ ^18^ ^]^ In light of this relation, we also found that the ratio of Co^2+^/Co^3+^ decreased firstly, and then increased simultaneously with the red shift of the A_1g_ peak in CoCr (Figure 4c). The initial decrease is relevant to the oxidation of Co^2+^ to Co^3+^. Since the average oxidation state of Co is determined to increase by the operando XAS, we speculate that the increasing ratio of Co^2+^/Co^3+^ is mainly due to the consumption of Co^3+^ to Co^4+^, which coincided with Co^3+/4+^ redox behavior difference between CoCr and as‐Co_3_O_4_ in CV curves (Figure S8, Supporting Information).^[^ ^21^ ^]^ We assume that the high covalency and flexibility of Co^3+^–O bonds rendered Co^3+/4+^ redox more facile, promoting the generation of the Co^4+^ active sites, which are consistent with the studies of XAS and XPS above. In addition, the degradation of Co_3_O_4_ depends on the growth of the hydrous oxide layer whose thickness is directly connected to the Co^3+/4+^ redox responses,^[^ ^8^ ^]^ explaining why the activity/stability tradeoff is broken in the CoCr catalyst.

We calculate the thermodynamic OER overpotential of CoCr based on the conventional adsorbates evolution mechanism (AEM) pathway by DFT (Figure 4d,e, and Figure S21, Supporting Information) since the lattice‐oxygen‐mediated mechanism (LOM) pathway studied by operando differential electrochemical mass spectroscopy (DEMS) measurements only accounted for a very small portion in our catalysts (Figure S22 and Table S7, Supporting Information). The CoCr catalyst showed an easy *O formation with a free energy change is about +1.38 eV, which was lower than that of as‐Co_3_O_4_ (+1.84 eV). This indicates a favorable extraction of electrons from oxygen agreeing well with the conclusion of PDOS. The potential determining step (PDS) on CoCr happened in the third step while as‐Co_3_O_4_ happened in the second step with a maximum free energy change of +1.64 and +1.84 eV, respectively. The corresponding theoretical overpotentials of CoCr and Co_3_O_4_ are 0.41 and 0.61 V, respectively.

## 3 Conclusion

The CoCr catalyst was successfully synthesized via a facile polymer‐assisted ultrasonic spraying and then annealing procedure on carbon paper. Comprehensive analyses using XPS, operando XAS, Raman spectra, and DFT calculations illuminated that the incorporation of Cr increases the oxidation state of Co and enhances the covalency and flexibility of Co–O bonds, which accelerates electron transfer and the variation of Co^3+^ to active high oxidation state Co^4+^, thereby positively influencing the OER process. So, both TOF and mass activity of CoCr were increased by eight times than those of as‐Co_3_O_4_. Impressively, the CoCr catalyst achieved a current density of 1.5 A cm^−2^ at only 2.17 V and remained stable at 500 mA cm^−2^ for 100 h in PEMWE. The optimization of sites of Co^3+^ deserves attention in design. This paves the way for the development of non‐precious metal electrocatalysts for PEMWE utilization.

## Conflict of Interest

The authors declare no conflict of interest.

## Author Contributions

B.Z. supervised this work. Q.Y. conceived and designed the experiments. Q.Y. synthesized the materials, measured the electrochemical performance, conducted the characterizations, and carried out corresponding operando spectroscopies. J.F. and. Y.L. performed the DFT calculations and analysis. W.S. and W.N. provided suggestions for article revision. Z.L., X.Y., and K.S. assisted in ICP measurements and Raman measurements. K.S. and L.X. participated in the operando XAS measurements. Y.L. contributed to the design of the synthesis scheme. Q.Y. and B.Z. wrote the manuscript. All of the authors discussed the results and commented on the manuscript.

## Supporting information

Supporting Information

## Data Availability

The data that support the findings of this study are available from the corresponding author upon reasonable request.
