# Supplementary material for: Chromium‐Induced High Covalent Co–O Bonds for Efficient Anodic Catalysts in PEM Electrolyzer
Source: Adv Sci (Weinh). 2024 Apr 22;11(25):2402356. doi: 10.1002/advs.202402356 (PMC11220634; doi:10.1002/advs.202402356)
Supplement: Supplementary file 1 — Supporting Information [file ADVS-11-2402356-s001.pdf]

ADVANCED SCIENCE
Open Access

Supporting Information

for *Adv. Sci.*, DOI 10.1002/advs.202402356

Chromium-Induced High Covalent Co–O Bonds for Efficient Anodic Catalysts in PEM Electrolyzer

*Qisheng Yan, Jie Feng, Wenjuan Shi, Wenzhe Niu, Zhuorong Lu, Kai Sun, Xiao Yang, Liangyao Xue, Yi Liu, Youyong Li and Bo Zhang**

Supporting Information for

Chromium-induced High Covalent Co-O Bonds for Efficient Anodic Catalysts in PEM Electrolyzer

Qisheng Yan[1], Jie Feng[2], Wenjuan Shi[1], Wenzhe Niu[1], Zhuorong Lu[1], Kai Sun[1], Xiao Yang[1], Liangyao Xue[1], Yi Liu[1], Youyong Li[2] and Bo Zhang[1]*

[1]*State Key Laboratory of Molecular Engineering of Polymers, Department of Macromolecular Science, Fudan University, Shanghai 200438, China.*

[2]*Institute of Functional Nano & Soft Materials (FUNSOM) and Jiangsu Key Laboratory for Carbon-Based Functional Materials & Devices, Soochow University, Suzhou 215123, China.*

**Correspondence: bozhang@fudan.edu.cn*

This file includes:

Experimental Section

Figure S1 to S22

Table S1 to S7

References

Experimental Section

Chemicals. $Co(NO_3)_2\cdot 6H_2O$ (99%) and $Cr(NO_3)_3\cdot 9H_2O$ (99%) were purchased from Aladdin. Ethanol (≥99.7%) and isopropanol (≥99.7%) were obtained from the Sinopharm Chemical Reagent Co., Ltd. As for carbon paper (CP), TGP-H-060 was provided by Toray and Sigracet 28BC was from the Fuel Cell Store. 5% Nafion® ionomer solution and Nafion 212 membrane were obtained from DuPont. 40% Pt/C catalyst was obtained from Johnson Matthey Company. IrO_2 catalyst was purchased from Umicore. Platinum plated titanium felt was purchased from NV Bekaert SA. Pure water was used in all processes. All chemicals were used without purification.

Synthesis of Cr-doped Co_3O_4 (CoCr) catalysts on carbon paper. The CoCr was synthesized according to the previous method with some modifications.[1] Typically, $Co(NO_3)_2\cdot 6H_2O$ (265 mg) and $Cr(NO_3)_3\cdot 9H_2O$ (40 mg) were dissolved in 980 µl of water, followed by addition of 20 µl 5% Nafion solution. The mixture was sonicated at room temperature for 30 min, and then was sprayed on carbon paper (TGP-H-060) on a hotplate. After film formation by drying, the precursors were calcined at 250 °C for 1 h and then annealed at 350 °C for 3 h in air to transform into oxides. The catalyst loading was controlled in 6 mg cm^{-2}. The as-synthesized Co_3O_4 (as-Co_3O_4) and other relevant catalysts were prepared as the same process with different ratios of $Co(NO_3)_2\cdot 6H_2O$ and $Cr(NO_3)_3\cdot 9H_2O$.

Characterizations. The X-ray diffraction (XRD) patterns of catalysts were performed with a point step of 0.02 degree by a Bruker D8A diffractometer using Cu Kα radiation. The surface morphology of catalysts was acquired by Scanning electron microscopy (SEM) using Bruker operated at 1.0 kV. The high-resolution transmission electron microscopy (HRTEM) images and corresponding energy-dispersive X-ray spectroscopy (EDS) elemental mapping were measured by a JEOL JEM-2100F transmission electron microscope with an Oxford energy disperse spectrometer. The X-ray photoelectron spectroscopy (XPS) was conducted using a Thermo Scientific K-Alpha+ equipped with an Al Kα X-ray source (1486.6 eV) for excitation. All peak energies were calibrated against the binding energy of the adventitious C 1s peak, which was fixed at 284.8 eV. The atomic composition of samples as well as Co and Cr leaching concentration were determined using an inductively coupled plasma optical emission spectrometry (ICP-OES) by Thermo icap 7400.

Operando differential electrochemical mass spectrometry (DEMS) studies with isotope labeling experiments were carried out on QAS 100 device from Linglu Instruments (Shanghai) Co. Ltd. A typical test was carried out in a three-electrode cell with 0.1 M $HClO_4$ as electrolyte. First, the catalysts were labelled with ^{18}O isotopes by performing 5 cyclic voltammetry (CV) cycles (0.6 - 1.4 V vs. Hg/Hg_2SO_4) at a scan rate of 5 mV s^{-1} in ^{18}O-labelled 0.1 M $HClO_4$. After removing the surface $H_2{}^{18}O$ and drying, the DEMS experiments were conducted 3 CV cycles under the same electrochemistry condition.

The ex-situ and *operando* X-ray Absorption spectroscopy (XAS) experiments of Co K-edge and O K-edge were performed at the 1W1B and 4B7B beamline of the Beijing Synchrotron Radiation Facility (BSRF). The ex-situ Co K-edge X-ray absorption fine structure (XAFS) data were obtained in transmission mode. The *operando* studies were conducted in fluorescent mode considering the electrolytes layer. A homemade three-electrode triangular cell was used to perform the *operando* studies.[2] The absorption energy of Co K-edge was calibrated by Co foil (7709 eV). E_0 of the standard foil was assigned by the second maximum of the first-derivative X-ray absorption near-edge structures (XANES) spectrum. All data were aligned according to the standard foil. The XAFS raw data were calibrated and normalized by the ATHENA program included in the IFEFFIT software package.[3] The curve-fitting analysis of the *operando* extended X-ray absorption fine structure (EXAFS) data in the R-space (1.0-3.0 Å, dR = 0, R-window = Hanning) and Fourier transforms in the k-space 3.0-12.2 $Å^{-1}$ (dk = 1, k-window = Hanning) were performed with the ARTEMIS program.

The *operando* Raman measurements were conducted with a Renishaw in Via Qontor Raman spectrometer in a homemade single cell with a 785 nm laser. Pt wire and Ag/AgCl (3.5 M KCl) were used as the counter and reference electrodes, respectively. The as-prepared Co-based catalysts were used as the working electrode with 0.5 M H_2SO_4 as the electrolyte.

Electrochemical measurements. Electrochemical studied were performed using a three-electrode system by an electrochemical workstation (Autolab PGSTAT204) equipped with a built-in electrochemical impedance spectroscopy (EIS) analyzer. A Pt

foil and Hg/Hg_2SO_4 were used as the counter and reference electrodes, respectively. 0.5 M H_2SO_4 was used as the electrolyte and the stirring rate was set at 300 r min^{-1}. Cyclic voltammetry (CV) test at 50 mV s^{-1} was conducted for 5 cycles before collecting linear scan voltammetry (LSV) at 5 mV s^{-1} for each sample. Electrochemical impedance spectroscopy data were recorded with a frequency scan range from 100 kHz to 0.005 Hz in a static solution at 1.57 V vs. RHE and then analyzed using the Voigt circuit model in Nova software package. All experiments were performed at ambient temperature (23 ± 2 °C), and the electrode potentials were converted to the RHE scale by the equation (1):

$$E(RHE) = E(Hg/Hg_2SO_4) + E(Hg/Hg_2SO_4) + 0.059pH = E(Hg/Hg_2SO_4) + 0.067 \quad (1)$$

IR correction. The correction was done using the equation (2):

$$E_{corrected} = E - iR \quad (2)$$

where $E_{Corrected}$ is the iR-corrected potential, E is the experimentally measured potential and R is the series resistance of measurement.

Electrochemical active surface area (ECSA) of each catalyst was derived from the electrochemical double-layer capacitances (C_{dl}) by recording the scan rate CV-dependence plot in a non-Faradaic region. The CV cycle potential window was 0.3 to 0.4 V vs. Hg/Hg_2SO_4, and the scan rates were 4, 6, 8, 10, and 12 mV s^{-1}. The difference of current density (Δj) between charging and discharging process at the average potential in the selected range was calculated from equation (3):

$$\Delta j = \frac{1}{2}\,(j_{charge} - j_{off\,charge}) \quad (3)$$

The slope of the linear fit was calculated as the C_{dl}. A specific capacitance of 40 μF cm^{-2} was used here.[4] The ECSA of the catalyst was calculated from the following equation (4):

$$ECSA = \frac{C_{dl}}{40\,\mu F\,cm^{-2}}\,cm^2_{ECSA} \quad (4)$$

Turnover frequency (TOF) determination. The number of active sites is calculated using the following equation[5] (5):

$$TOF = \frac{j \times A}{4 \times F \times m} \quad (5)$$

where j (mA cm^{-2}) is the current density measured at 1.67 V vs. RHE, A is the geometric area of carbon paper electrode (0.25 cm^2), F is the Faraday constant (96,485 C mol^{-1}), m is the mole number of metal site, and the number 4 refers to four electrons per O_2. The TOFs were calculated assuming that all the metal ions were catalytically active, therefore representing the lower limit of the activity.

The proton exchange membrane water electrolysis (PEMWE) test was carried out on the DC power source (PWR401L, KIKUSUI Trading Co., Ltd.). The anodic catalyst (CoCr) loading was controlled at 4.8 mg cm^{-2} by the same synthesis. The ink of Pt/C catalyst was prepared by ultrasonically blending catalyst with Nafion solution and isopropanol. The Nafion® ionomer-to-catalyst ratio was 0.3. The cathodic side was prepared at 80 °C by spraying Pt/C catalyst ink, and the loading was controlled at 0.3 mg_{Pt} cm^{-2}. IrO_2 was used as reference and the loading was controlled at 2 mg cm^{-2}. Platinum plated titanium felt and carbon paper (Sigracet 28BC) were taken as the current collector on the anodic and cathodic sides, respectively. They were closely assembled against the titanium plates in the single cell. The active area was 5 cm^{-2} and the 80 °C pure water was fed to the anode.

Computational details. We perform density functional theory (DFT) calculations in the present work by the Viena Ab initio Simulation Package (VASP),[6] and employ the generalized gradient approximation of Perdew-Burke-Ernzerhof (GGA-PBE) to describe the exchange-correlation energy and electronic exchange.[7] We set the cutoff energy to 400 eV, and utilize the energy convergence criterion of 10^{-4} eV and the force convergence criterion of 0.03 eV Å $^{-1}$. The Γ-centered k-point grid is set to 3 × 3 × 3 and 1 × 1 × 1 for bulk calculations and slab calculations, respectively, and for all slabs, a vacuum distance of 15 Å was added along the z direction.

The oxygen evolution reaction (OER) reaction under acidic conditions contains four intermediate steps as follows:[8]

$$H_2O + * \rightarrow OH^* + (H^+ + e^-) \tag{6}$$

$$OH^* \rightarrow O^* + (H^+ + e^-) \tag{7}$$

$$H_2O + O^* \rightarrow OOH^* + (H^+ + e^-) \tag{8}$$

$$OOH^* \rightarrow O_2 + * + (H^+ + e^-) \tag{9}$$

where * represents an active site on the clean surface, and *OH, *O, and *OOH denote hydroxyl, oxo-, and hydroperoxide intermediates adsorbed on the surface, respectively.[9]

The Gibbs free-energy change (ΔG) for every OER elementary step is given as the difference between the initial and final states:

$$\Delta G=\Delta E+\Delta ZPE-T\Delta S \tag{10}$$

where ΔE denotes the energy difference between the reaction reactants and products, ΔZPE represents changes in the zero-point energy, and ΔS represents changes in entropy.

The limiting potential of the OER is defined as the change in Gibbs free energy in the potential determining step (PDS), namely

$$U_{limiting}=max\{\Delta G_i\} \tag{11}$$

and the overpotential of the OER reaction can be written as

$$\eta=\frac{U_{limiting}}{e}-1.23 \tag{12}$$

The d band center was calculated by following formula:

$$\epsilon_d=\frac{\int \epsilon\rho(\epsilon)d\epsilon}{\int \rho(\epsilon)d\epsilon} \tag{13}$$

where ϵ and $\rho(\epsilon)$ denote energy and density of states, respectively.

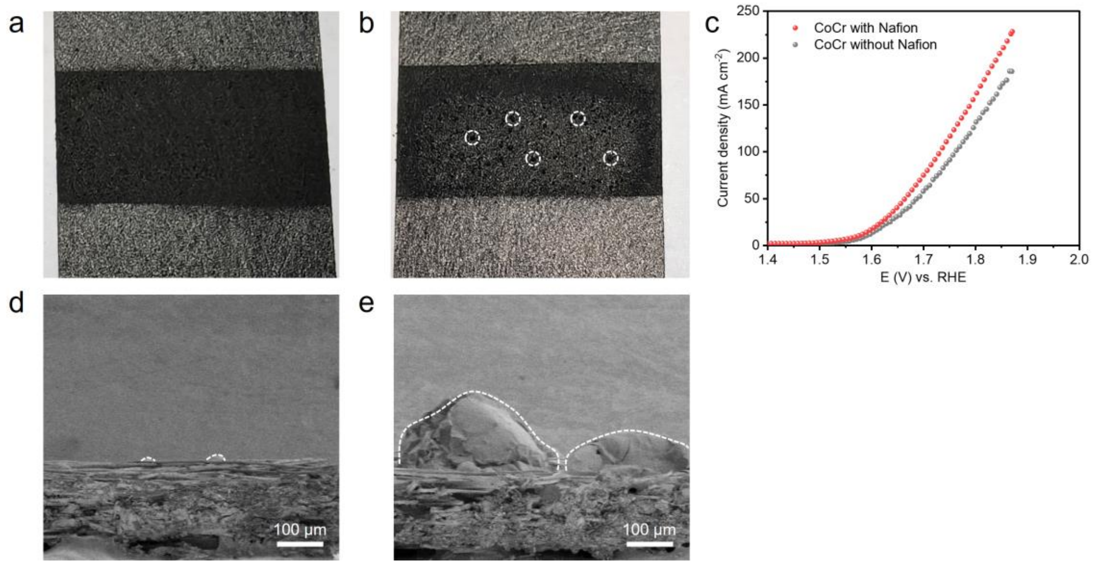

Figure S1. The different morphology of CoCr catalysts synthesized a, d) with Nafion and b, e) without Nafion on carbon paper. a, b) Photographs of catalysts loaded on carbon paper. c) LSV curves of CoCr synthesized with Nafion and without Nafion in 0.5 M H_2SO_4 solution without iR correction. d, e) SEM images of cross section of carbon paper. The profiles depicted by dashed lines indicate catalyst aggregation. It is clear that the polymer-assisted method can contribute to uniform catalyst layer, avoid sintering and improve OER catalytic activity.

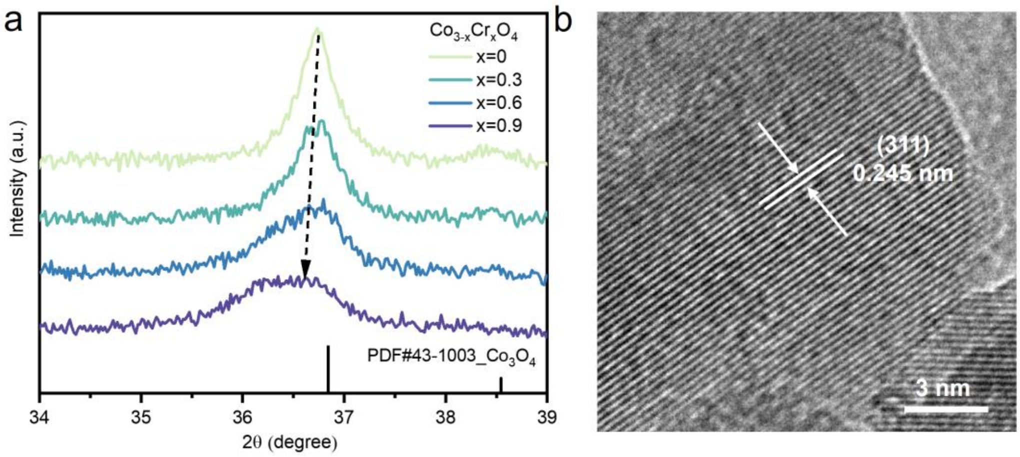

Figure S2. a) XRD patterns of different Cr doping contents of Co_3O_4 at (311) facet. b) lattice spacing of as-Co_3O_4 catalyst. The peak (311) shifted to lower degree with doping Cr, suggesting an expanding interplanar spacing trend, which coincided with the result of HRTEM.

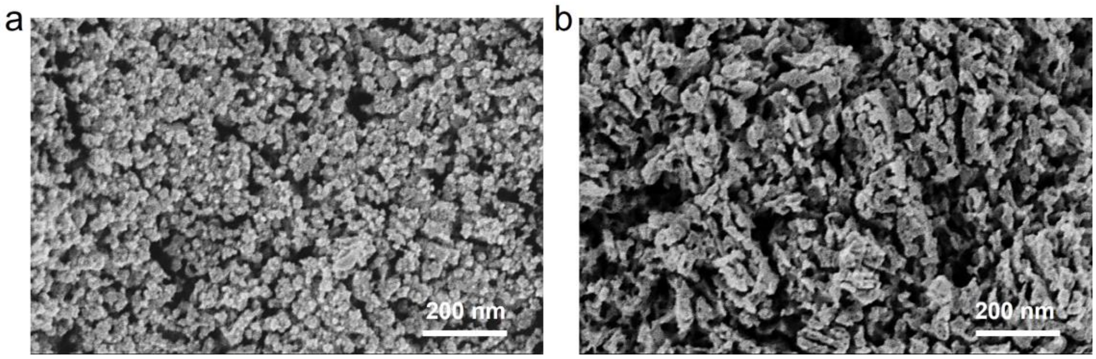

Figure S3. SEM images of CoCr (a) and as-Co_3O_4 (b) catalysts on carbon paper substrates. CoCr catalyst exhibits smaller nanoparticles and more nanopores, which is beneficial for mass transfer.

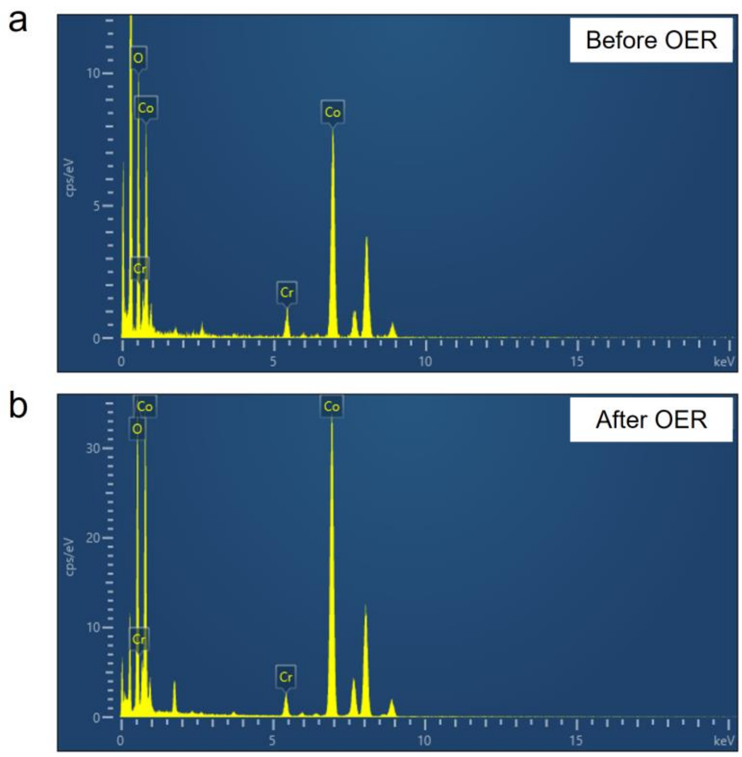

Figure S4. TEM-EDS spectra of the CoCr catalyst a) before and b) after OER tested in 0.1 M $HClO_4$ solution.

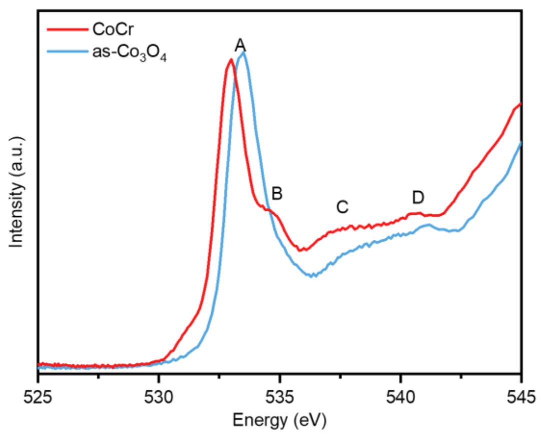

Figure S5. O K-edge XANES spectra of CoCr and as-Co_3O_4 catalysts. The main peaks (marked A, B, C and D) in the region 533-543 eV arise from the transition of O 1s to higher-energy states of O p character hybridized with the metal 4d or 4sp band.[10] In contrast to as-Co_3O_4, the peaks show a left-shift and a new feature B appears in CoCr due to introducing Cr and decreasing content of Co.[10a, 10c] The enhancing intensity of feature C and D suggest that Cr incorporation can hinder distortion of the local structure around the Co or O atom, and improve the Co site symmetry.[10b]

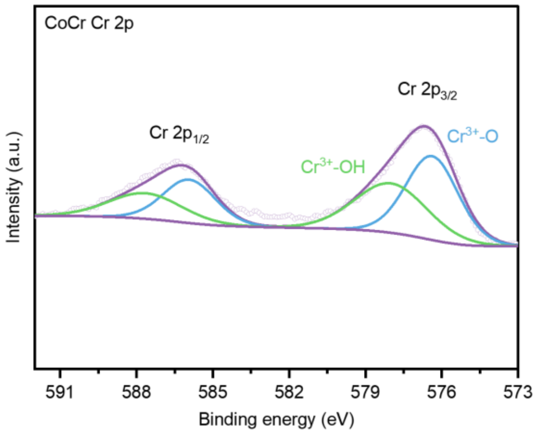

Figure S6. XPS spectra of Cr 2p in CoCr catalyst before OER. The Cr $2p_{3/2}$ was fitted by two peaks at 577.6 and 576.2 eV, which can be attributed to Cr^{3+}-OH and Cr^{3+}-O, respectively.

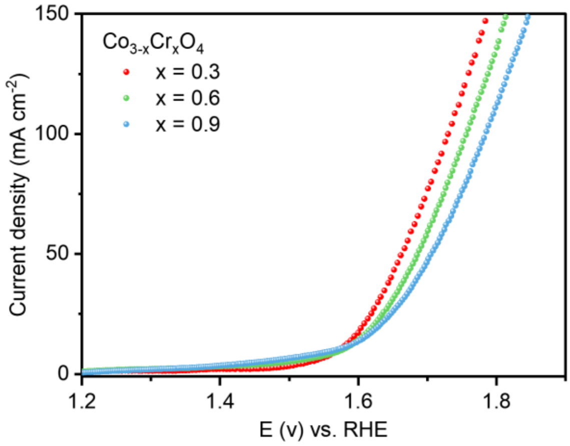

Figure S7. LSV curves of different Cr doping contents in 0.5 M H_2SO_4 solution without iR correction.

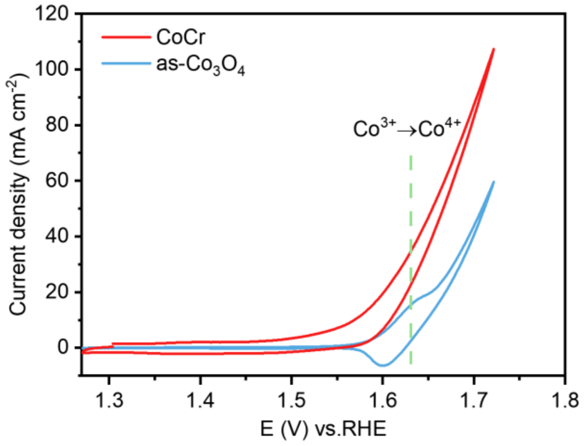

Figure S8. CV curves of CoCr and as-Co_3O_4 catalysts in 0.5 M H_2SO_4 solution. The CoCr catalyst shows no obvious $Co^{3+/4+}$ redox feature.

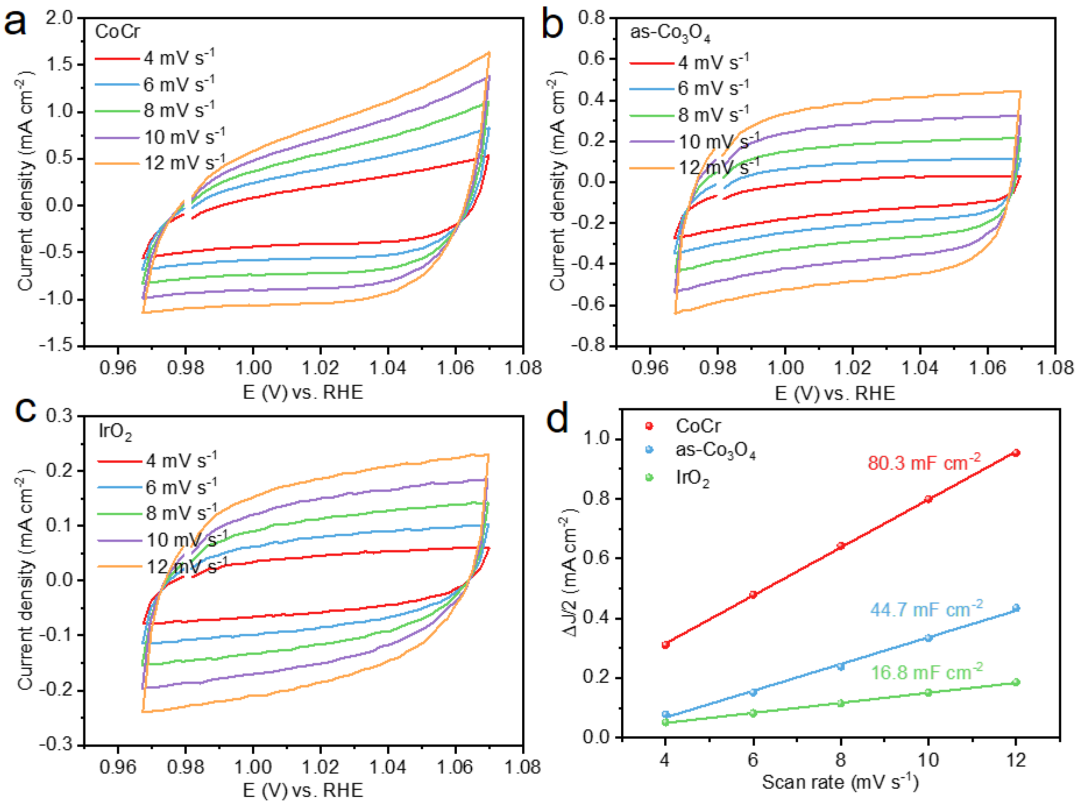

Figure S9. ECSA characterization. Cyclic voltammetry plots of a) CoCr, b) as-Co_3O_4 and c) IrO_2 catalysts at 0.97-1.07 V vs. RHE at scan rates from 4 to 12 mV s^{-1} in 0.5 M H_2SO_4 solution. d) The current density of catalysts based on scan rate and their corresponding linear fittings (solid lines) and calculated C_{dl}. The obtained ECSA are 2007, 1118 and 420 cm^2 for CoCr, as-Co_3O_4 and IrO_2 catalysts, respectively.

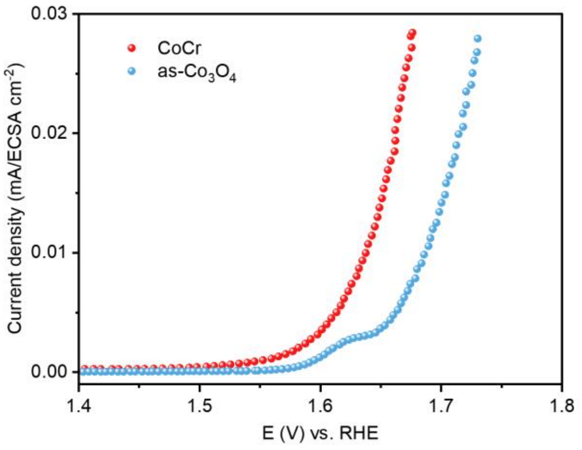

Figure S10. ECSA-normalized polarization curves. ECSA-normalized OER polarization curves of CoCr and as-Co_3O_4 catalysts on carbon paper substrates in 0.5 M H_2SO_4 solution.

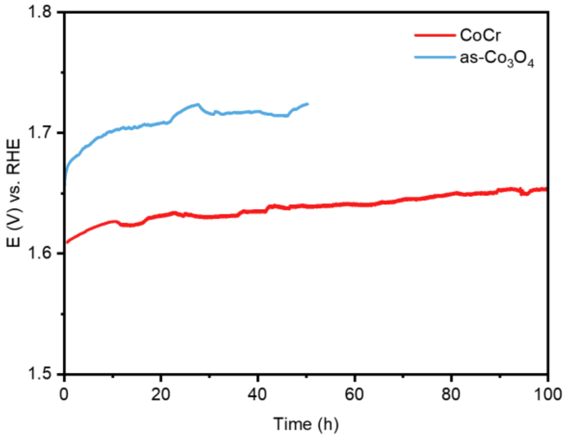

Figure S11. Chronopotentiometry curves of catalysts measured at a constant current density of 10 mA cm^{-2} in 0.1 M $HClO_4$.

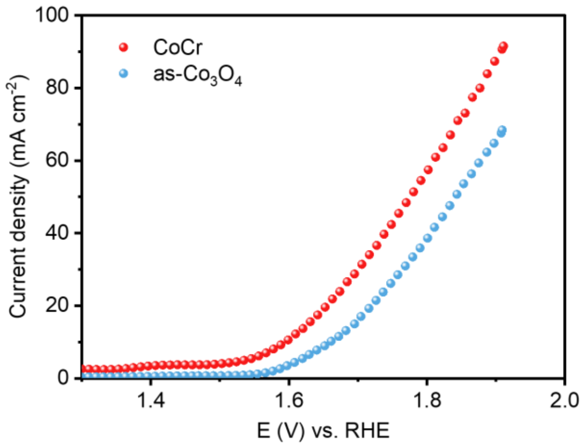

Figure S12. LSV curves of CoCr and as-Co_3O_4 catalysts in 0.1 M $HClO_4$ solution without iR correction. The overpotential of CoCr reaching 10 mA cm^{-2} is 360 mV, still lower than that of as-Co_3O_4 (430 mV).

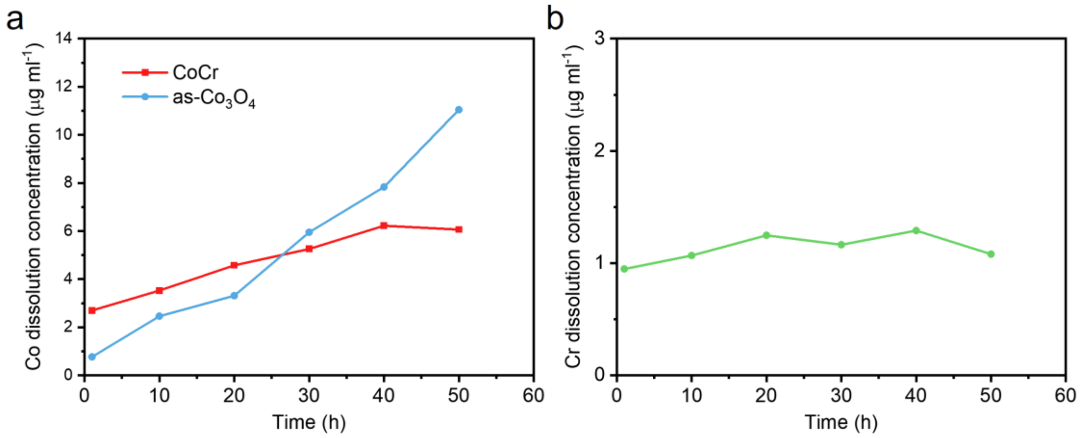

Figure S13. ICP-OES measurements of Co and Cr dissolution concentration vs. time in 0.1 M $HClO_4$ solution at current density of 10 mA cm^{-2}. We took out 1 mL from 50 mL testing electrolyte during the stability test and the electrolyte was replenished at the same amount.

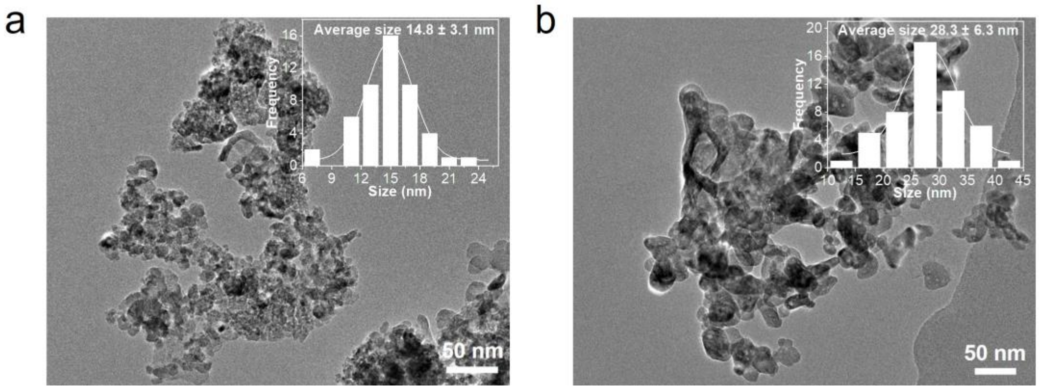

Figure S14. TEM images of the a) CoCr and b) as-Co_3O_4 catalysts after OER tested in 0.1 M $HClO_4$ solution. Insets are nanoparticle size statistics. There was no significant difference about shape and size between pre reaction and post reaction both in CoCr and as-Co_3O_4 catalysts.

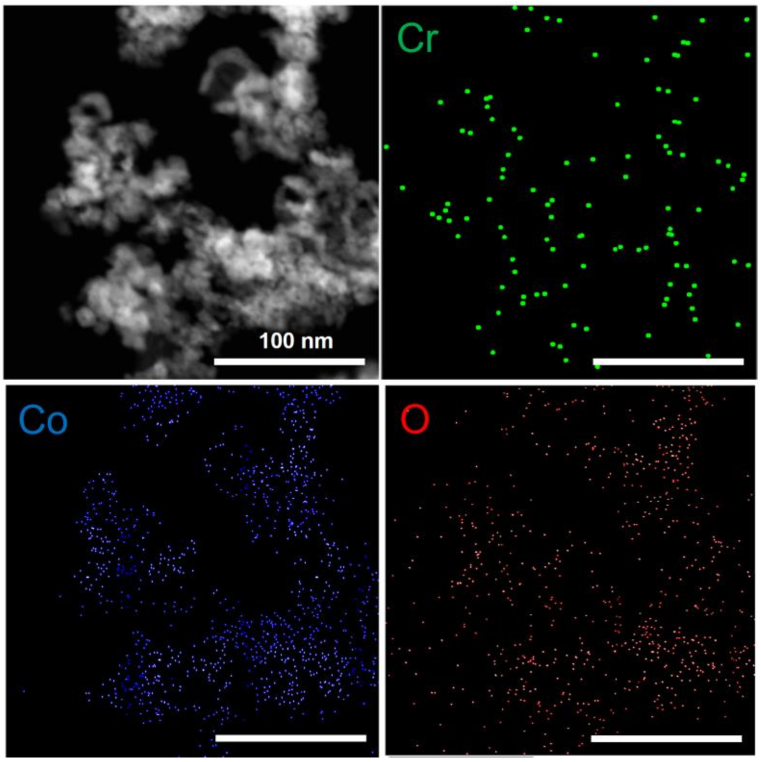

Figure S15. TEM-EDX elemental mapping of the CoCr catalyst after OER tested in 0.1 M $HClO_4$ solution.

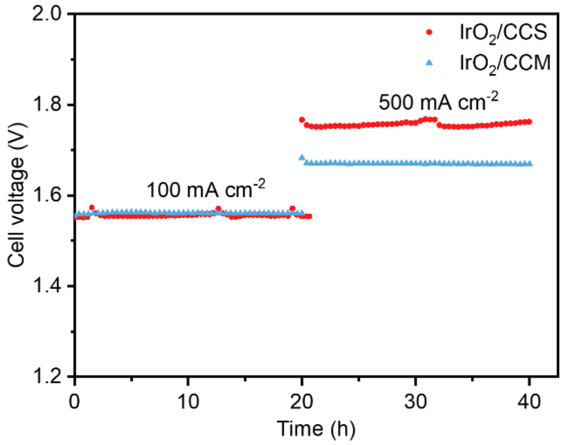

Figure S16. Chronopotentiometry curves of the PEMWE using IrO_2 as reference operated at 100 mA cm^{-2} and 500 mA cm^{-2} at 80 °C by different MEA structures. IrO_2/CCS means that catalysts are loaded on the carbon paper while IrO_2/CCM suggests that catalysts are sprayed directly on the Nafion 212 membrane.

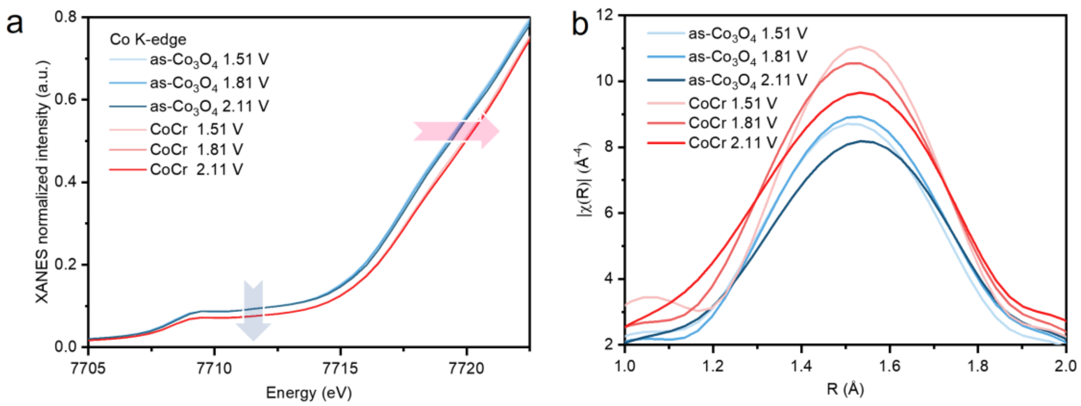

Figure S17. *Operando* XAS analysis. a) XANES spectra of Co K-edge and b) R-space EXAFS spectra. The average oxidation state of Co was enhancing followed by increasing the potential and maintained at higher value in CoCr than as-Co_3O_4 samples all the time. The symmetry of Co also kept higher in CoCr.

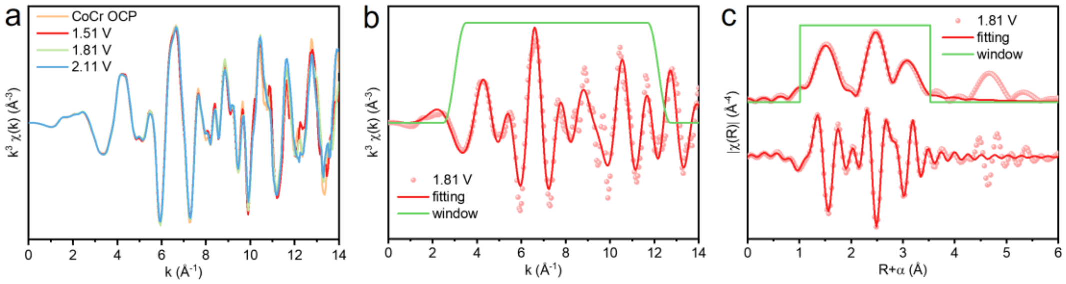

Figure S18. EXAFS characterizations at Co K-edge of CoCr catalyst. a) k^3-weighted EXAFS functions at Co K-edge of CoCr catalyst at open circuit potential (OCP) and different applied potential. An example of EXAFS curve fitting b) in k-space and c) in R-space at 1.81 V vs. RHE. All data were not phase-corrected.

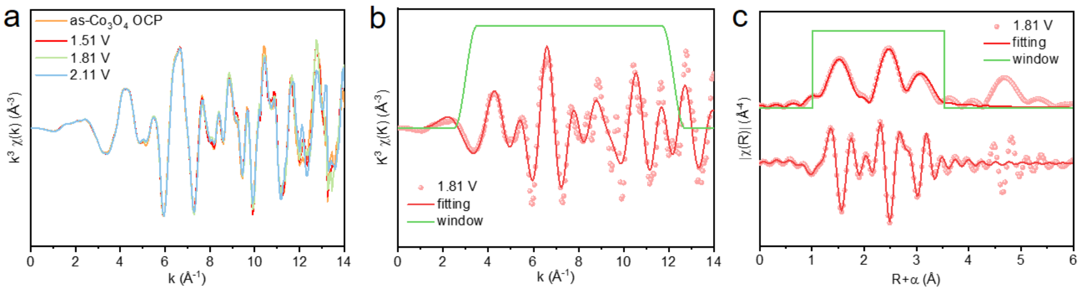

Figure S19. EXAFS characterizations at Co K-edge of as-Co_3O_4 catalyst. a) k^3-weighted EXAFS functions at Co K-edge of as-Co_3O_4 catalyst at OCP and different applied potential. An example of EXAFS curve fitting b) in k-space and c) in R-space at 1.81 V vs. RHE. All data were not phase-corrected.

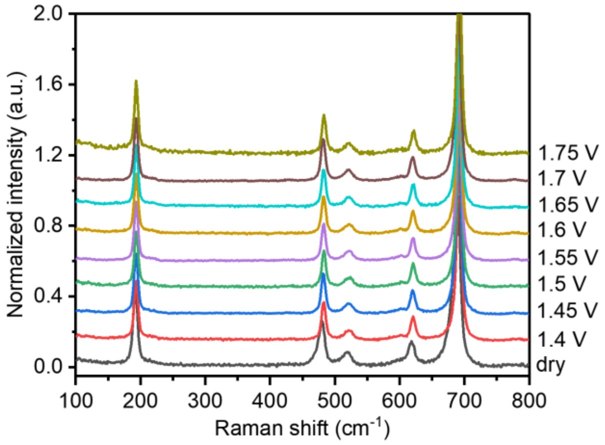

Figure S20. *Operando* Raman spectra of as-Co_3O_4 at various constant potentials (vs. RHE) without iR correction (increased from 1.4 to 1.75 V).

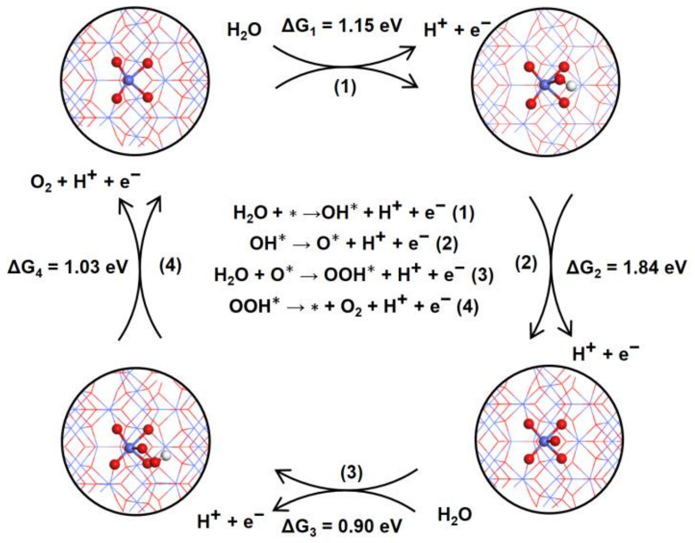

Figure S21. Simulated OER reaction pathways of as-Co_3O_4 with the intermediates adsorbed including OH*, O*, and OOH* on the (311) surface. Reaction 2 is the potential-limiting step. The blue, red and white balls represent Co, O and H, respectively.

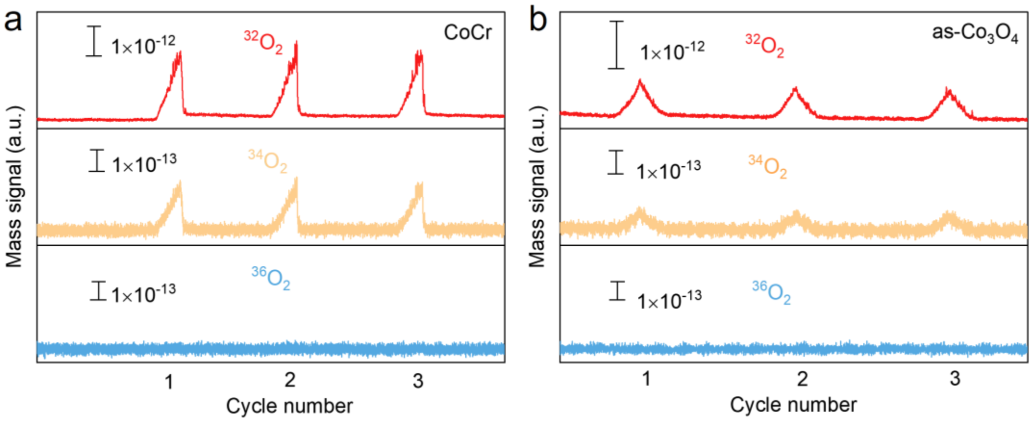

Figure S22. DEMS signals of O_2 products for a) CoCr and b) as-Co_3O_4 labelled in the electrolyte using $H_2{}^{18}O$ as the solvent during three times of CV cycles in the potential range of 1.27-2.07 V vs. RHE, with a scan rate of 5 mV s^{-1}.

Table S1. The ratios of Co and Cr metal in the CoCr catalyst determined by ICP and TEM-EDS, respectively, before and after OER tested in 0.1 M $HClO_4$ solution.

	TEM-EDS	ICP
Before OER	8.4 : 1	9.2 : 1
After OER	14.6 : 1	26.5 : 1

Table S2. Fitting parameters[a)] for CoCr using Co K-edge EXAFS.

Sample	Path[b)]	N	R (Å)	$\sigma^2 \times 10^{-3}$ ($Å^2$)[c)]	R factor[d)]
CoCr 1.51V	Co-O	3.7 (± 0.0)	1.93 (± 0.00)	2.5 (± 0.0)	
	Co-Co1	3.4 (± 0.0)	2.87 (± 0.01)	4.7 (± 0.6)	0.014
	Co-Co2	5.4 (± 1.3)	3.38 (± 0.01)	6.7 (± 2.0)	
CoCr 1.81V	Co-O	3.5 (± 0.1)	1.92 (± 0.00)	2.5 (± 0.0)	
	Co-Co1	3.9 (± 0.1)	2.87 (± 0.00)	4.9 (± 0.0)	0.003
	Co-Co2	3.7 (± 0.2)	3.38 (± 0.00)	3.9 (± 0.0)	
CoCr 2.11V	Co-O	3.4 (± 0.0)	1.91 (± 0.00)	2.8 (± 0.0)	
	Co-Co1	3.9 (± 0.0)	2.86 (± 0.00)	4.6 (± 0.4)	0.009
	Co-Co2	3.3 (± 0.6)	3.37 (± 0.00)	3.0 (± 1.2)	
CoCr (OCP)	Co-O	3.6 (± 0.4)	1.93 (± 0.00)	3.2 (± 1.4)	
	Co-Co1	3.3 (± 0.7)	2.86 (± 0.00)	4.2 (± 1.5)	0.012
	Co-Co2	5.4 (± 1.6)	3.38 (± 0.01)	6.7 (± 2.3)	

[a)] XAS data process and analysis were conducted utilizing ATHENA and ARTEMIS software. All the spectra were calibrated, background-subtracted and normalized in conformity to the protocols in ATHENA documentation. S_0^2 was fixed as 1.0 for cobalt oxide. ΔE_0 was refined as a globe fit parameter, returning a value of (2 ± 2) eV. Data fitting range for Co: 3.0 ≤ k ($Å^{-1}$) ≤ 12.2 and 1.0 ≤ R (Å) ≤ 3.5. All the fits were performed in the R space with K-weight of 3. All the potentials are vs RHE.
[b)] The distances for Co-O and Co-Co paths were calculated from the crystal structure of Co_3O_4.
[c)] The Debye-Waller factors (σ^2) were constrained after the last fitting to decrease the number of variables.
[d)] R factors were calculated to describe the fitting quality (≤ 0.02). All fitting results were satisfied.

Table S3. Fitting parameters for as-Co_3O_4 using Co K-edge EXAFS.

Sample	Path	N	R (Å)	$\sigma^2 \times 10^{-3}$ (Å^2)	R factor
as-Co_3O_4 1.51V	Co-O	2.7 (± 0.2)	1.92 (± 0.00)	1.8 (± 0.0)	
	Co-Co1	2.6 (± 0.2)	2.87 (± 0.01)	3.6 (± 0.0)	0.010
	Co-Co2	3.8 (± 0.9)	3.38 (± 0.01)	4.9 (± 1.7)	
as-Co_3O_4 1.81V	Co-O	2.8 (± 0.0)	1.92 (± 0.00)	2.1 (± 0.4)	
	Co-Co1	3.3 (± 0.5)	2.87 (± 0.01)	5.0 (± 1.2)	0.006
	Co-Co2	3.3 (± 0.8)	3.38 (± 0.01)	4.4 (± 1.6)	
as-Co_3O_4 2.11V	Co-O	2.5 (± 0.2)	1.93 (± 0.00)	1.6 (± 1.0)	
	Co-Co1	4.1 (± 0.7)	2.88 (± 0.01)	6.7 (± 1.7)	0.011
	Co-Co2	2.2 (± 0.2)	3.39 (± 0.01)	1.7 (± 0.0)	
as-Co_3O_4 (OCP)	Co-O	2.7 (± 0.2)	1.92 (± 0.00)	1.8 (± 0.7)	
	Co-Co1	2.7 (± 0.3)	2.87 (± 0.00)	3.7 (± 0.8)	0.004
	Co-Co2	3.8 (± 0.6)	3.38 (± 0.00)	4.7 (± 1.1)	

Table S4. The extracted R_s and R_{ct} values of the catalytic OER on the catalysts on carbon paper at an overpotential of 340 mV in 0.5 M H_2SO_4 solution.

Samples	R_s (Ω)	R_{ct} (Ω)	CPE
CoCr	3.57	22.7	0.95
as-Co_3O_4	3.28	221	0.97
IrO_2	4.17	13.0	0.76

Table S5. The OER activity and stability of selected electrocatalysts in acid

Catalyst	Overpotential (mV)[a)]	Electrolyte	Stability (h) @ j (mA cm^{-2})	References
**CoCr**	**333**	**0.5 M H_2SO_4**	**100@500[b)]**	**This work**
Co_3O_4/CeO_2	347	0.5 M H_2SO_4	50@10	[11]
Co_3O_4@C/CP	370	0.5 M H_2SO_4	86.8@100	[12]
$Co_{3-x}Ba_xO_4$	278	0.5 M H_2SO_4	110@10	[4]
CoLaMn	335	0.5 M H_2SO_4	100@210[b)]	[5]
$Mn_{7.5}O_{10}Br_3$	295	0.5 M H_2SO_4	300@100[b)]	[13]
Co_3O_4/FTO	570	0.5 M H_2SO_4	12@10	[14]
$Cu_{1.5}Mn_{1.5}O_4$:F	325	0.5 M H_2SO_4	24@16	[15]
γ-MnO_2/CP	428	1.0 M H_2SO_4	12@100[b)]	[16]
Ag–Co_3O_4	470	0.5 M H_2SO_4	10@10	[17]
Mo-Co_9S_8@C	370	0.5 M H_2SO_4	24@10	[18]

[a)] The overpotential reaching 10 mA cm^{-2}.
[b)] Tested in PEMWE.

Table S6. Comparisons of the water electrolysis metrics of CoCr with other reported catalysts in membrane electrode assembly.

Catalyst	Current density ($mA\ cm^{-2}$)	Stability (h)	Voltage[a)] (V)	References
**CoCr**	**700**	**20**	**1.96**	**This work**
**CoCr**	**500**	**100**	**1.80**	**This work**
**CoCr**	**100**	**500**	**1.67**	**This work**
S-RuO_2/ATO	500	40	1.55	[19]
In-RuO_2/graphene	100	350	1.53	[20]
Y_2MnRuO_7	200	24	1.51	[21]
$Mn_{7.5}O_{10}Br_3$	100	300	1.8	[13]
$La_2Sr_2Ni_1Fe_2$[b)]	500	40	1.8	[22]
B-MOF-Zn-Co[b)]	200	300	1.78	[23]
LaMnCo	210	100	1.65	[5]
$W_{0.2}Er_{0.1}Ru_{0.7}O_{2-\delta}$	100	120	1.6	[24]
$Nd_{0.1}RuO_x$/CC	10	50	1.5	[25]
LaSrCo oxide[b)]	300	140	1.85	[26]

[a)] The voltage is associated with the selected current density.
[b)] The catalysts are applied in AEMWE.

Table S7. Mass spectroscopy peak area of different catalysts labelled by $H_2{}^{18}O$ as the solvent.

Sample	Scan number	$^{32}O_2$ area ($\times10^{-11}$ a.u.)	$^{34}O_2$ area ($\times10^{-11}$ a.u.)	Area ratio ($^{34}O_2$: $^{32}O_2$)	LOM content (%)	LOM content average (%)
	1	7.618	1.264	0.166	14.23	
as-Co_3O_4	2	6.318	1.144	0.181	15.33	14.78
	3	6.731	1.168	0.174	14.79	
	1	15.313	2.077	0.136	11.94	
CoCr	2	14.740	1.867	0.127	11.24	11.81
	3	14.058	1.961	0.139	12.24	

References

[1] A. Li, S. Kong, C. Guo, H. Ooka, K. Adachi, D. Hashizume, Q. Jiang, H. Han, J. Xiao, R. Nakamura, *Nat. Catal.* **2022**, 5, 109.

[2] Y. Wen, P. Chen, L. Wang, S. Li, Z. Wang, J. Abed, X. Mao, Y. Min, C. T. Dinh, P. D. Luna, R. Huang, L. Zhang, L. Wang, L. Wang, R. J. Nielsen, H. Li, T. Zhuang, C. Ke, O. Voznyy, Y. Hu, Y. Li, W. A. Goddard Iii, B. Zhang, H. Peng, E. H. Sargent, *J. Am. Chem. Soc.* **2021**, 143, 6482.

[3] B. Ravel, M. Newville, *J. Synchrotron Radiat.* **2005**, 12, 537.

[4] N. Wang, P. Ou, R. K. Miao, Y. Chang, Z. Wang, S.-F. Hung, J. Abed, A. Ozden, H.-Y. Chen, H.-L. Wu, J. E. Huang, D. Zhou, W. Ni, L. Fan, Y. Yan, T. Peng, D. Sinton, Y. Liu, H. Liang, E. H. Sargent, *J. Am. Chem. Soc.* **2023**, 145, 7829.

[5] L. Chong, G. Gao, J. Wen, H. Li, H. Xu, Z. Green, J. D. Sugar, A. J. Kropf, W. Xu, X.-M. Lin, H. Xu, L.-W. Wang, D.-J. Liu, *Science* **2023**, 380, 609.

[6] a) G. Kresse, J. Furthmüller, *Phys. Rev. B* **1996**, 54, 11169; b) G. Kresse, J. Furthmüller, *Comput. Mater. Sci.* **1996**, 6, 15; c) J. Hafner, *J. Comput. Chem.* **2008**, 29, 2044.

[7] a) P. E. Blöchl, *Phys. Rev. B* **1994**, 50, 17953; b) J. P. Perdew, K. Burke, M. Ernzerhof, *Phys. Rev. Lett.* **1996**, 77, 3865.

[8] J. Rossmeisl, Z. W. Qu, H. Zhu, G. J. Kroes, J. K. Nørskov, *J. Electroanal. Chem.* **2007**, 607, 83.

[9] I. C. Man, H.-Y. Su, F. Calle-Vallejo, H. A. Hansen, J. I. Martínez, N. G. Inoglu, J. Kitchin, T. F. Jaramillo, J. K. Nørskov, J. Rossmeisl, *ChemCatChem* **2011**, 3, 1159.

[10] a) X. Long, P. Yu, N. Zhang, C. Li, X. Feng, G. Ren, S. Zheng, J. Fu, F. Cheng, X. Liu, *Nanomaterials* **2019**, 9, 577; b) D. Chen, J. Zhong, X. Wu, Z. Wu, N. Mironova-Ulmane, A. Kuzmin, A. Marcelli, *Spectrochim. Acta, Part A* **2008**, 70, 458; c) C.-L. Chen, C.-L. Dong, K. Asokan, G. Chern, C. L. Chang, *Solid State Commun.* **2018**, 272, 48.

[11] J. Huang, H. Sheng, R. D. Ross, J. Han, X. Wang, B. Song, S. Jin, *Nat. Commun.* **2021**, 12, 3036.

[12] X. L. Yang, H. N. Li, A. Y. Lu, S. X. Min, Z. Idriss, M. N. Hedhili, K. W. Huang, H. Idriss, L. J. Li, *Nano Energy* **2016**, 25, 42.

[13] S. Pan, H. Li, D. Liu, R. Huang, X. Pan, D. Ren, J. Li, M. Shakouri, Q. Zhang, M. Wang, C. Wei, L. Mai, B. Zhang, Y. Zhao, Z. Wang, M. Graetzel, X. Zhang, *Nat. Commun.* **2022**, 13, 2294.

[14] J. S. Mondschein, J. F. Callejas, C. G. Read, J. Y. C. Chen, C. F. Holder, C. K. Badding, R. E. Schaak, *Chem. Mater.* **2017**, 29, 950.

[15] P. P. Patel, M. K. Datta, O. I. Velikokhatnyi, R. Kuruba, K. Damodaran, P. Jampani, B. Gattu, P. M. Shanthi, S. S. Damle, P. N. Kumta, *Sci. Rep.* **2016**, 6.

[16] A. L. Li, H. Ooka, N. Bonnet, T. Hayashi, Y. M. Sun, Q. K. Jiang, C. Li, H. X. Han, R. Nakamura, *Angew. Chem. Int. Ed.* **2019**, 58, 5054.

[17] K. L. Yan, J. F. Qin, J. H. Lin, B. Dong, J. Q. Chi, Z. Z. Liu, F. N. Dai, Y. M. Chai, C. G. Liu, *J. Mater. Chem. A* **2018**, 6, 5678.

[18] L. G. Wang, X. X. Duan, X. J. Liu, J. Gu, R. Si, Y. Qiu, Y. M. Qiu, D. E. Shi, F. H. Chen, X. M. Sun, J. H. Lin, J. L. Sun, *Adv. Energy Mater.* **2020**, 10, 1903137.

[19] B. Huang, H. Xu, N. Jiang, M. Wang, J. Huang, L. Guan, *Adv. Sci.* **2022**, 9, 2201654.

[20] Y. Wang, X. Lei, B. Zhang, B. Bai, P. Das, T. Azam, J. Xiao, Z.-S. Wu, *Angew. Chem. Int. Ed.* **2024**, 63, e202316903.

[21] D. Galyamin, J. Torrero, I. Rodríguez, M. J. Kolb, P. Ferrer, L. Pascual, M. A. Salam, D. Gianolio, V. Celorrio, M. Mokhtar, D. Garcia Sanchez, A. S. Gago, K. A. Friedrich, M. A. Peña, J. A. Alonso, F.

Calle-Vallejo, M. Retuerto, S. Rojas, *Nat. Commun.* **2023**, 14, 2010.
[22] D. Chen, Y. S. Park, F. Liu, L. Fang, C. Duan, *Chem. Eng. J.* **2023**, 452, 139105.
[23] X. Lin, X. Li, L. Shi, F. Ye, F. Liu, D. Liu, *Small*, 2308517.
[24] S. Hao, M. Liu, J. Pan, X. Liu, X. Tan, N. Xu, Y. He, L. Lei, X. Zhang, *Nat. Commun.* **2020**, 11, 5368.
[25] L. Li, G. Zhang, J. Xu, H. He, B. Wang, Z. Yang, S. Yang, *Adv. Funct. Mater.* **2023**, 33, 2213304.
[26] L. Osmieri, Y. He, H. T. Chung, G. McCool, B. Zulevi, D. A. Cullen, P. Zelenay, *J. Power Sources* **2023**, 556, 232484.
